# *In situ* occurrence and protection of crop wild relatives in Italian sites of natura 2000 network: Insights from a data-driven approach

**DOI:** 10.3389/fpls.2022.1080615

**Published:** 2022-12-22

**Authors:** Lorenzo Raggi, Cecilia Zucchini, Daniela Gigante, Valeria Negri

**Affiliations:** Dipartimento di Scienze Agrarie, Alimentari e Ambientali (DSA3), Università degli Studi di Perugia, Perugia, Italy

**Keywords:** CWR, crop wild relatives, *in situ* conservation, natura 2000 network, protected areas, GIS, land use land cover

## Abstract

Aim of this work is to evaluate the *in situ* status of different crop wild relative species in Italy by analysing the geographic distribution of their populations and to suggests possible strategies to improve their future conservation. The work has been focused on different species of the *Allium, Beta, Brassica, Secale* and *Triticum* genera that are of priority at European and global levels for the economic importance of the related crops, the level of threat, as well as the potential for use. Using information available in the Italian National Geoportal, geographical distribution and the overall percentage of populations occurring in Natura 2000 sites was initially analysed. In addition, due to the economic importance of the genus and species distribution in Italy, *Brassica glabrescens*, *B. insularis, B. macrocarpa*, *B. montana*, *B. procumbens*, *B. rupestris, B. villosa* were the object of additional analyses based on more detailed occurrence data, retrieved from multiple databases, and including land cover/land use and *in situ* and *ex situ* density analyses. Geographical distribution data were retrieved for 1,996 *in situ* populations belonging to 60 crop wild relative species: *Allium* (43)*, Brassica* (11)*, Triticum* (4), *Beta* (1) and *Secale* (1). Percentages of population occurring in Natura 2000 sites are quite different when the different species are considered; this also applies to *Brassica* species in most need of protection. Results of land cover/land use analysis showed that *Brassica* populations outside Natura 2000 areas mainly occur in anthropized sites while those within Natura 2000 mainly in sites characterised by natural and seminatural conditions. Areas where genetic reserves could be instituted and that could be the target of future *Brassica* resources collection missions are also suggested. Based on a large dataset of punctual geographical distribution data of population occurrences across the territory, this research shows that, in Italy, crop wild relatives *in situ* are in a quite precarious condition especially when species in most need of protection are considered. Our data also highlight the role of Natura 2000 Network in favouring *in situ* protection of these precious resources in Europe.

## Introduction

Biodiversity is presently at risk from multiple threats and in need to be urgently protected and maintained for the future. The Mediterranean basin is an important biodiversity hotspot with about 25,000 plant species (Cuttelod et al., 2008), of which about 13,000 are endemic (Myers et al., 2000). The Mediterranean basin is also one of the primary centres of origin of important crops and/or secondary centre of diversity of other relevant crops domesticated in different places. Approximately 80% of the Euro-Mediterranean flora consists of wild harvested plants, crop species and their wild relatives; in other words, more than three-quarters of the plant species in the region have a current or potential use (Ford-Lloyd et al., 2011). As such they are important Plant Genetic Resources (PGR), *i.e.* not only a fundamental biodiversity component and heritage of evolution in nature, but also a component of biodiversity which is essential to respond to the new stresses in the agricultural environment, including those produced by climate changes, and to secure the present and future welfare and food security of human kind. However, many of these species, also including some of those with current or potential use, are at risk (Bilz et al., 2011; Kell et al., 2011) and considered in need of protection and/or monitoring by national and international conservation policies such as the Bern Convention (Council of Europe, 1979) and the Habitats Directive 43/92/EEC (European Commission, 1992).

Due to the relevance of biodiversity for current and future generations, in the last decades the European Union (EU) has promoted different protection measures that also effected PGR. Indeed, since the above mentioned Habitats Directive 43/92/EEC was established in 1992, EU and its Member States have made commitments and set clear goals to stop biodiversity loss. Legal frameworks, such as the Birds and Habitats Directives; policies, such as the EU Biodiversity Strategy (European Commission, 2011); and financial mechanisms, such as the LIFE programme (Yougova, 2021) were put in place to support the achievement of these goals. All these activities ensued in the creation of the Natura 2000 Network (Evans, 2012), the World’s largest network of protected areas covering about 18.5% of the European land area and almost 10% of the total EU marine area (EEA, 2020). However, as a consequence of the insufficient advance towards achieving biodiversity conservation targets − as certified by the “Report on the status and trends in 2013 - 2018 of species and habitat types protected by the Birds and Habitats Directives” (European Commission, 2020) − the EU has recently adopted the European Green Deal that represents a strong commitment by the European Commission to legally binding restoration of degraded habitats, including their biodiversity and also provides financial support for the implementation of such activities (European Commission, 2019). Aligning with the Green Deal objectives, the recently adopted Biodiversity Strategy for 2030 (European Union, 2020) provides further policy context and opportunities for biodiversity conservation and restoration. On this regard, it should be finally noted that the EU also contributed to the development and subscribed to international agreements and conventions, such as the UN Sustainable Development Goals (UNITED NATIONS, 2015) or the Convention on Biological Diversity (CBD, 1992), that lead global initiatives for halting biodiversity loss (for a review on the topic see Hermoso et al., 2022).

A crop wild relative (CWR) is defined as a wild plant *taxon* that has an indirect use derived from its, relatively close, genetic relationship to a crop. This relationship can be defined based either on the “Gene Pool” or on the “Taxon Group” concept. According to the former, a CWR is any species included in the primary or secondary Gene Pool of a crop, as defined by Harlan and Wet (1971); the latter refers to the broader, more inclusive, approach proposed by Maxted et al. (2006) where all the wild species belonging to the same genus of any crop should be considered as CWR. Being a massive source of genetic variability and of traits potentially useful for crop improvement, CWR have a high socio-economic value and are identified among the main PGR. Since the very beginning of “formal” plant breeding, from 1940 and 1950s and even more by the 1960s and 1970s, CWR were routinely used by breeders to improve major crops (Meilleur and Hodgkin, 2004) and their use is likely to progressively increase in the future to face crop yields constraints. As a matter of fact, they are extremely valuable in adapting crop varieties to changing disease pressures, farming practices, market demands and climatic conditions; as such, they can increase the adaptive capacity of agricultural systems around the world (Keesing et al., 2010; Dempewolf et al., 2017; Sharma et al., 2021). However, the ecosystems in which CWR live are becoming increasingly unstable, making many populations at risk of extinction.

It is commonly acknowledged that the inter- and intra-specific diversity, as well as the habitat diversity of wildlife, is under threat of irremediable loss (Cardinale et al., 2012; Ceballos et al., 2015; Janssen et al., 2016; Leigh et al., 2019; Chase et al., 2020; Orsenigo et al., 2021). This is the reason why it is urgent to develop a systematic strategy for their monitoring and effective conservation, to assure their future survival. It is now important that national, regional, and global strategies for complementary CWR *in situ* and *ex situ* conservation be developed and implemented and that priority CWR species and their respective location sites be identified (Ford-Lloyd et al., 2011).

The most appropriate and effective protection strategy for CWR appears to be the *in situ* conservation. In fact, preserving CWR populations in their natural habitat allows them to continue to evolve and generate new genetic diversity, favouring their adaptation to the changing climate and environmental conditions (CBD, 1992; Heywood and Dulloo, 2005; Stolton et al., 2006). However, *in situ* conservation alone is not sufficient to safeguard diversity being populations in the wild often object of stresses and pressures that can dramatically reduce number of individuals and mine populations survival. It is therefore important to support the *in situ* with *ex situ* approach mainly realised through seed sample conservation in genebanks or plants conservation in living collections. To maximise the level of conserved diversity the two approaches should be integrated (Maxted et al., 1997; Brush, 2004; Phillips et al., 2016; Schwartz et al., 2017). The *ex situ* conservation is also relevant allowing the access to genetic resources easier. However, Castañeda-Álvarez et al. (2016) showed that 95% of global priority CWR are poorly represented in genebanks; considering 441 priority CWR present in the Fertile Crescent, Zair et al. (2021) reported that 30% are totally absent from the current gene banks collections while 57% have less than 10 accessions. This is mainly due to elements of technical and managerial nature (Hunter and Heywood, 2011): the storage conditions, set for the main crops, are not always suitable for CWR which can present recalcitrant seeds, dormancy as well as germination problems.

As for the *in situ* approach, a system of protected areas is the basis for preservation strategies of most countries (Maxted et al., 1997; Heywood and Dulloo, 2005; Iriondo et al., 2008; Vincent et al., 2012) including Europe (Maxted, 2003). Indeed, the localization of CWR populations inside a protected area could guarantee a certain level of protection, although passive, *i.e.* without active population management by conservation agencies (Maxted et al., 2008). It is anyway to underline that to achieve a sufficient protection level, occurrence and population census monitoring, indispensable activities requiring specialized skills, should be included among conservation objectives of protected areas with consequent strengthening and enforcing of the management plans. It is also noteworthy that many populations of CWR of different species of interest are currently occurring outside protected areas (Heywood and Dulloo, 2005): road and field edges as well as cultivated fields may host CWR populations.

To generate national and international conservation plans, the first step is to create and maintain updated dedicated inventories of species. These inventories serve as the basis for an analysis of their patterns of distribution, consistency and conservation status, level of threat, current conservation actions and identification of priority sites in need of conservation (Maxted et al., 2007). Using a pragmatic approach based on species value, native status and need of protection and/or monitoring, a new prioritized list of wild plants of socio-economic interest has been recently drawn up for Italy by Ciancaleoni et al. (2021); similar lists have been also developed for other countries (El Mokni et al., 2022 and references therein). Once the high-priority species have been defined, it is important to examine the distribution of their populations across the territory. Unfortunately, geographic distribution of species is often available only at a coarse geographic scale (*e.g.* occurrence or not at administrative regional level) as also emphasized by Orsenigo et al. (2021), while a precise information on punctual occurrence, sites location, and census of CWR populations need to be retrieved for the implementation of effective conservation activities (Ciancaleoni et al., 2021).

To support the formulation and implementation of appropriate conservation measures, the goal of this work is to evaluate a basic component of the present CWR *in situ* conservation status in Italy by evaluating the fine geographic distribution of their populations. Based on the analysis of a large set of data retrieved from different databases, the research has been focused on CWR species of *Allium, Beta, Brassica, Secale* and *Triticum* genera which are priority at European and global level for the economic importance of the related crops, the level of threat, as well as the potential for use (Castañeda-Álvarez et al., 2016; Vincent et al., 2019). Some of the species of *Allium, Brassica*, and *Triticum* have been also identified as most in need of protection in Italy in the work of Ciancaleoni et al. (2021) and therefore deserve special conservation efforts. Given the commitment of EU countries in protecting plant genetic resources under the international policies and the role that the Natura 2000 Network can play in achieving such objectives (Rubio Teso et al., 2020; Raggi et al., 2022), the proportion of populations located in sites of the Network was also evaluated and discussed. Finally, *Brassica* CWR species in most need of protection were used as a case study: sites hosting populations of these species were the object of territory as well as land cover and land use analysis aiming at depicting the main differences existing among protected and non-protected sites and their consequences on CWR *in situ* conservation opportunities.

## Materials and methods

### CWR distribution data source

The work is focused on the different CWR species − defined according to the approach of Maxted and colleagues (2006) mentioned in the Introduction section − belonging to the genera *Allium, Beta, Brassica, Secale* and *Triticum* that are considered of priority at European and global levels. From June to August 2020, all the georeferenced occurrence data of populations of CWR species belonging to these genera available in the Italian ‘National Biodiversity Network’ database were retrieved through the cartographic viewer. The viewer is accessible on the Italian ‘National Geoportal’ (http://geoviewer.nnb.isprambiente.it/mapreacter) that is part of a shared data management system created to support the ‘National Strategy for Biodiversity’, which in turn is promoted by the ‘Ministry of Ecological Transition’ (*Ministero della Transizione Ecologica*, MiTE), the Italian Ministry in charge of protecting nature. Collated by the *Istituto Superiore per la Protezione e la Ricerca Ambientale* (ISPRA), data are archived in a database built on: MiTE’s list of ‘Rare and endangered species of Italian flora’, ‘Red and blue lists of the Italian flora’ (Pignatti et al., 2001), the ‘Floristic-vegetational database’ of the Italian Botanical Society and the ‘Atlas of the vascular flora of Lazio’. Only those populations recorded in the Italian ‘National Biodiversity Network’ database, and holding precise georeferred coordinates, have been included in the present study; coming from an unqualified source, data from iNaturalist were not considered (Rubio Teso et al., 2020). Even if the database cannot be yet considered an exhaustive source of all the floristic occurrences in Italy, it hosts a huge amount of georeferred occurrences of plant species populations and is the only national-scaled tool providing an overview of their distribution across the whole Italian territory.

Retrieved data were initially organised in a database where, for each record of presence of a certain CWR population (database entry), the following fields were annotated: i) taxonomy and nomenclature including genus, species and author, ii) geographic coordinates (latitude and longitude) and administrative regions, iii) presence (or not) in a site of the Natura 2000 Network and iv) when valid, type, code, name and extension of the site. Taxonomic nomenclature was harmonised according to the Portal to the Flora of Italy (https://dryades.units.it/floritaly/index.php) that is based on Bartolucci et al. (2018) and later updates.

### Distribution of *Allium, Beta, Brassica, Secale* and *Triticum* CWR species

Once homogenised and organised data were imported into QGIS software, v3.16.15-Hannover (QGIS Development Team, 2021) specifying the geographic reference system WGS84 (EPSG: 4326) not projected, compliant with the LAT/LONG DD format. For the definition of national border, the polygonal shape file (scale of 1:1 000 000) of the administrative borders of Italy was used (Eurostat, 2019). Distribution of the populations through the Italian territory, and with respect to the Natura 2000 Network, was graphically elaborated by genus and species.

### Distribution of *Allium, Brassica* and *Triticum* CWR species most in need of protection

For each target genus, a subset of species most in need of protection was compiled including only those listed as ‘A’ category in Ciancaleoni et al. (2021). For these species, the occurrence within Natura 2000 Network was evaluated at the administrative region level and regional distributions were compared with those reported in the *Portal to the Flora of Italy* (https://dryades.units.it/floritaly/) which organizes the nomenclatural and distributive data deriving from the most recent checklists of native and alien plants of Italy (Bartolucci et al., 2018; Galasso et al., 2018, and later updates). This comparison was only performed to validate the obtained CWR species distribution in the different Italian administrative regions. which was based on georeferenced populations occurrence data.

### Fine-scale characterisation of sites hosting *Brassica* priority species, a case study

#### Data collection and quality control

Considering their distribution on the national territory and the importance of related crops, for the seven *Brassica* CWR species in most need of protection, georeferenced data of population occurrence were also retrieved from two international biodiversity databases: the Global Biodiversity Information Facility (GBIF) and Genesys. GBIF is an international network and data infrastructure funded by the world’s governments aimed at providing open access to chorological data of all types of life on Earth that includes data from genebanks, botanic gardens, museums, and universities. Genesys is a database holding information on *ex situ* accessions conserved in genebanks worldwide; it is also fed by numerous national and international data providers.

Retrieved data were initially checked for crop nomenclature consistency and Latin names were homogenised according to the international project COL. As suggested by Rubio Teso and colleagues (2020), different filters were then applied to create a high-quality dataset where occurrences belonging to the following classes were removed: i) cultivated materials; ii) not recorded in Italy; iii) with missing or low-quality geographical coordinates (*i.e.* ≤ 2 decimal digits or stated error > 500 meters); iv) dated before 1970 and, v) duplicates, keeping those more recent and with more available information. For GBIF data, records with the following characteristics were also deleted: i) with major known issue (*i.e.* invalid basis of record, fuzzy institution match, country coordinate mismatch) and, ii) coming from unreliable or unqualified sources (*i.e.* iNaturalist).

#### Distribution and protection status

Data were imported into QGIS software specifying the same geographic reference system and definition of national border as described above. A spatial consistency verification was then carried out and occurrences with the following characteristics were eliminated: i) placed outside national borders or in the country centroid and ii) placed in the sea; the few records near enough to the coasts (<1 km) to be considered as evidently misplaced due to small inaccuracies of the data, were manually repositioned along the coast. Results were graphically elaborated using the same GIS software.

Filtered data were organised in two databases:

- *Brassica in situ* database, including the records from GBIF, Genesys and the National Biodiversity Network
- *Brassica ex situ* database, only including the records from Genesys.

*Brassica in situ* database records (*i.e.* sites hosting *Brassica* species most in need of protection) were then characterised using different cartographic sources by means of the ‘Vector overlay’ function in QGIS software. The function extracts the portions of features from the input layer that overlap features in the overlay layer by placing geographically referenced points (georeferenced records in the *Brassica in situ* database) within the limits of any of the polygons composing the geospatial vector (cartography sources). Features that share the same spatial extent are then output in a table that inherits attributes from both layers. Results of the analysis were included in the *Brassica in situ* database. The following geographical layers were used as cartographic sources: i) Biogeographical regions (updated to January 2016), retrieved from the European Environment Agency (https://www.eea.europa.eu/data-and-maps/figures/biogeographical-regions-in-europe-2) that are adopted as an official tool for the EU Habitats Directive (92/43/EEC) (European Commission, 1992) and Birds Directive (79/409/EEC 1979) and later updates (European Commission, 1979) and ii) Natura 2000 Network (updated to March 2020), retrieved from the European Environment Agency (https://www.eea.europa.eu/data-and-maps/data/natura-11), that accounts for delineations used in the EU Habitats Directive (92/43/EEC) and for those of the EMERALD Network set up under the ‘Convention on the Conservation of European Wildlife and Natural Habitats’ (*i.e.*, Bern Convention) (Council of Europe, 1979). Also in this case, species distributions were then compared with those reported in the *Portal to the Flora of Italy* while percentages of populations in Natura 2000 sites were compared with the results here obtained only using data from the Italian ‘National Geoportal’.

#### Land cover and land use

The “Vector overlay” function was also used to get insight on Land Cover and Land Use (LCLU) of the sites hosting populations of the species *Brassica insularis* Moris.*, B. montana* Pourr*., B. rupestris* Raf. and *B. villosa* Biv. the only species with a sufficient number of *in situ* occurrence datapoints to justify the analysis (see results).

To the purpose both the Corine Land Cover (CLC) (2018) (status layer) and the ESA CCI Land Cover v2.0.7 (ESA Land Cover CCI project team and Defourny, 2019) cartographic layers were used. Corine information on different territories is organised in 5 principal classes, furtherly divided for a total of 44 categories. Developed in the frame of ESA CCI Land Cover project, based on the UN Land Cover Classification System (LCCS) developed by the United Nations (UN) Food and Agriculture Organization (FAO), ESA CCI Land Cover v2.0.7 gives information on the land use according to 22 primary and 14 secondary use classifications (ESA, 2017). To clearly show LCLU differences between sites hosting CWR populations located inside or outside the Natura 2000 sites, only the principal Corine classes were here considered: 1. Artificial surfaces, 2. Agricultural areas, 3. Forest and seminatural areas, 4. Wetlands, and 5. Water bodies. In addition, to facilitate the comparison of LCLU results based on Corine and ESA CCI cartographies, categories of the latter were summarised as detailed in **Table 1**. LCLU was performed by species and results summarised using histograms; being not relevant considering either absolute or relative number of sites both by- and overall-species “Wet lands” (Corine) and “Bare areas” (ESA CCI) categories were not displayed.

**Table 1:** Corine main categories and corresponding “ESA CCI groups of categories” employed in this study with the list of included ESA CCI categories.

Corine main category	ESA CCI group of categories	Included ESA CCI categories and corresponding number
1. Artificial surfaces	Urban areas	Urban areas (190)
2. Agricultural areas	Cropland, rainfed + Mosaic cropland	Cropland, rainfed (10)
		Cropland, rainfed - herbaceous cover (11)
		Cropland, rainfed -Tree or shrub cover (12)
		Mosaic cropland (>50%)/natural vegetation (tree, shrub, herbaceous cover) (<50%) (30)
3. Forest and seminatural areas	Natural vegetation	Mosaic natural vegetation (tree, shrub, herbaceous cover) (>50%)/cropland (<50%) (40)
		Tree cover, broadleaved, deciduous, closed to open (>15%) (60)
		Tree cover, needleleaved, evergreen, closed to open (>15%) (70)
		Tree cover, mixed leaf type (broadleaved and needleleaved) (90)
		Mosaic tree and shrub (>50%)/herbaceous cover (<50%)(100)
		Shrubland (120)
		Grassland (130)
		Sparse vegetation (tree, shrub, herbaceous cover) (<15%) (150)
		Shrub or herbaceous cover, flooded, fresh/saline/brakish water (180)

#### *In situ* vs *ex situ* density analyses

To identify areas rich of *Brassica* populations, *in situ* and *ex situ Brassica* database records distribution was compared by means of a density analysis performed using QGIS software as described in Raggi et al. (2022). The grid of cells (10 km side), georeferenced in EPSG: 3035 (Lambert Azimuthal Equal Area), was obtained from the standard ones available at the European Commission (EC) website (European Environment Agency, 2017). Cells including ≥1 CWR population record were identified, the number of records *per* cell calculated using the “Count Points in Polygon” tool. Analysis results were graphically elaborated using the same GIS software.

## Results

### Distribution of *Allium, Beta, Brassica, Secale* and *Triticum* CWR species

**Figure 1:**
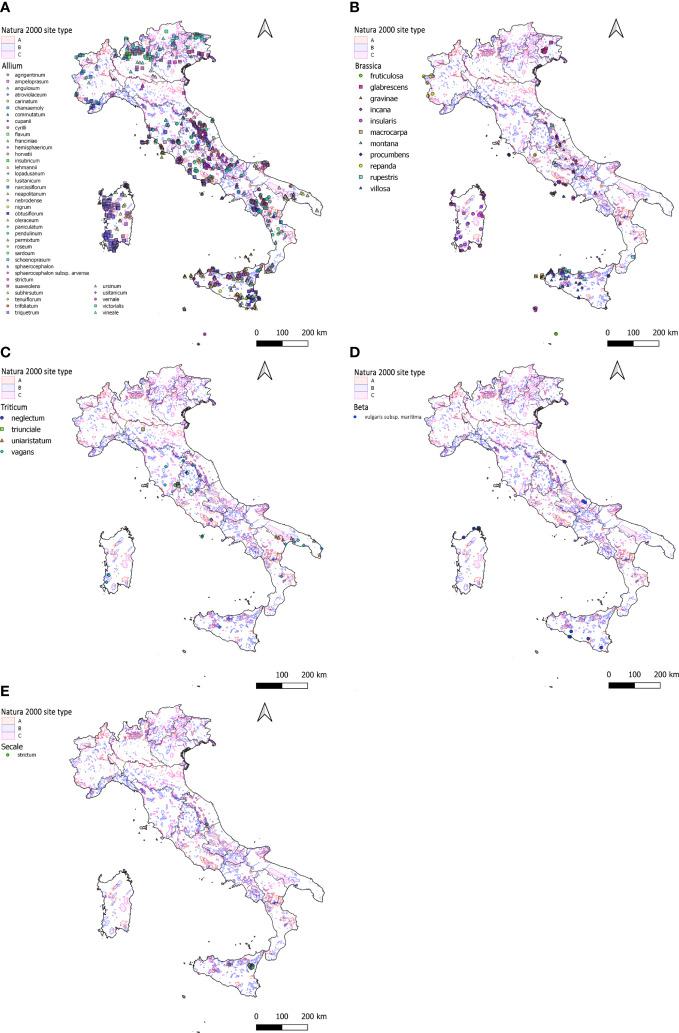
Distribution across Italy of the 1,996 populations of the CWR species belonging to *Allium* **(A)**, *Brassica* **(B)**, *Triticum* **(C)**, *Beta* **(D)** and *Secale* **(E)** as from data available in the Italian National Geoportal. **(A–E)** species and the different types of Natura 2000 sites are highlighted using different colours as reported in the figure legend. Letters associated to the Natura 2000 sites are as follows: “A” = designed “Special Protection Area” (SPA); “B” = “proposed Sites of Community Importance” (pSCI), “Sites of Community Importance” (SCI) or “Special Area of Conservation” (SAC); “C” = pSCI/SCI/SAC is the same as designated SPA.

Information on 1,996 populations of 60 different CWR species of the genera *Allium*, *Beta*, *Brassica*, *Secale* and *Triticum* were successfully retrieved from the Italian National Geoportal (**Table 2**); 1,344 populations, mainly of *Allium* species, occur in Natura 2000 sites that correspond to the 67% of the total *circa* (**Figure 1**).

**Table 2:** List of the 60 analysed *taxa*; names are listed according to the alphabetic order.

#	Species name	#	Species name
1	*Allium agrigentinum* Brullo & Pavone	31	*Allium schoenoprasum* L.
2	*Allium ampeloprasum* L.	32	*Allium sphaerocephalon* L.
3	*Allium angulosum* L.	33	*Allium sphaerocephalon* L. subsp. *arvense* (Guss.) Arcang.*
4	*Allium atroviolaceum* Boiss.	34	*Allium strictum* Schrad.
5	*Allium carinatum* L.	35	*Allium suaveolens* Jacq.
6	*Allium chamaemoly* L	36	*Allium subhirsutum* L.
7	*Allium commutatum* Guss.	37	*Allium tenuiflorum* Ten.
8	*Allium cupanii* Raf.	38	*Allium trifoliatum* Cirillo
9	*Allium cyrilli* Ten.	39	*Allium triquetrum* L.
10	*Allium flavum* L.	40	*Allium ursinum* L.
11	*Allium franciniae* Brullo & Pavone	41	*Allium vernale* Tineo
12	*Allium hemisphaericum* (Sommier) Brullo	42	*Allium victorialis* L.
13	*Allium horvatii* Lovrić	43	*Allium vineale* L.
14	*Allium insubricum* Boiss. & Reut. ex Reut.	44	*Beta vulgaris* L. subsp. *maritima* (L.) Arcang.**
15	*Allium lehmannii* Lojac.	45	*Brassica fruticulosa* Cirillo
16	*Allium lopadusanum* Bartolo, Brullo & Pavone	46	*Brassica glabrescens* Poldini
17	*Allium lusitanicum* Lam.	47	*Brassica gravinae* Ten.
18	*Allium narcissiflorum* Vill.	48	*Brassica incana* Ten.
19	*Allium neapolitanum* Cirillo	49	*Brassica insularis* Moris
20	*Allium nebrodense* Guss.	50	*Brassica macrocarpa* Guss.
21	*Allium nigrum* L.	51	*Brassica montana* Pourr.
22	*Allium obtusiflorum* Redouté	52	*Brassica procumbens* (Poir.) O.E.Schulz
23	*Allium oleraceum* L.	53	*Brassica repanda* (Willd.) DC.
24	*Allium pallens* L.	54	*Brassica rupestris* Raf.
25	*Allium paniculatum* L.	55	*Brassica villosa* Biv.
26	*Allium pendulinum* Ten.	56	*Secale strictum* (C.Presl) C.Presl
27	*Allium pentadactyli* Brullo, Pavone & Spamp.	57	*Triticum neglectum* (Req. ex Bertol.) Greuter
28	*Allium permixtum* Guss.	58	*Triticum triunciale* (L.) Raspail
29	*Allium roseum* L.	59	*Triticum uniaristatum* (Vis.) K.Richt.
30	*Allium sardoum* Moris	60	*Triticum vagans* (Jord. & Fourr.) Greuter

*Syn. *Allium arvense* Guss. **Syn. *Beta maritima L.*

Nomenclature is according to the Portal to the Flora of Italy (https://dryades.units.it/floritaly/index.php). Being recently reinterpreted as subspecies, *Allium sphaerocephalon* L. subsp. *arvense* (Guss.) Arcang. and *Beta vulgaris* L. subsp. *maritima* (L.) Arcang. are the only two *taxa* whose subspecies is reported.

With 43 different CWR species and 1,687 populations records retrieved, *Allium* is the genus with the highest number of occurrences in Italy among those here considered. 1,144 of these populations (68%) are located in Natura 2000 sites and distributed as follows: 17% within “Special Protection Areas” (SPAs) (‘A type’ sites), 66% within Special Conservation Areas (SACs) and a few (1%) in “Sites of Community Importance” (SCI) (‘B type’ sites) and 16% within SPAs coinciding with proposed Community Interest (pSCIs) (‘C type’ sites). Populations of *Allium* genus are distributed in a quite homogeneous way throughout the national territory (**Figure 1A**), present in all the different Italian administrative regions they are predominant in i) southern Italy, especially in Sicily (384 populations) and Sardinia islands (275) and ii) central Italy, especially in Lazio (202 populations). Populations are mainly within Natura 2000 sites in Campania (96%, n=119), Apulia (86%, n=132), Lazio (84%, n= 275), Abruzzo (77%, n=57) and Sicily (69%, n=384). On the other hand, lower percentages regarded Friuli-Venezia Giulia (43%, n=28), Trentino-Alto Adige (40%, n=15) and Calabria (34%, n=32) (**Figure 1A**).

Data on 215 populations of 11 different *Brassica* CWR species were successfully retrieved from the National Geoportal. 146 (68%) are in Natura 2000 sites: 57% in SACs, 18% in SICs, about 16% in SPAs while only a few (8%) in ‘type C’ sites. Populations are distributed along the Piedmont Alpine belt, in the Friulian plain, along the Central Apennine belt extended to the Gulf of Gaeta, and in Sicily and Sardinia (**Figure 1B**). Considering the Italian administrative regions, populations are mainly found in Sicily (67) followed by Lazio, Sardinia and Marche (39, 38 and 26, respectively). A high percentage of populations in Natura 2000 sites characterises Abruzzo and Molise (100%), Marche (92%), Lazio (87%) and Sardinia (79%) while about half of the populations are in Natura 2000 sites in Sicily (**Figure 1B**).

Data on 46 populations belonging to 4 different species were successfully retrieved for *Triticum*. Unevenly distributed between Central and Southern Italy, about 40% of these populations occur within the Natura 2000 Network: 55% in ‘type B’, 28% in ‘type A’ and 17% in ‘type C’ sites, respectively. The region with the highest number of observations is Apulia (12), followed by Lazio and Umbria (10 and 9) respectively; the genus is also found in Marche, Sardinia, Tuscany, Sicily, Basilicata, Calabria and Emilia Romagna with a rather small number of populations (**Figure 1C**). In Lazio, 60% of the populations are within the Natura 2000 Network while lower percentages characterise the other regions including Apulia (33%) (**Figure 1C**).

With 15 populations recorded, *Beta vulgaris* subsp. *maritima* is the only *taxon* of *Beta* genus considered in this study. Populations of this subspecies are mainly located in the major islands and along the Adriatic coast of Central Italy (**Figure 1D**). Overall, 53% of the populations are within Natura 2000 sites and similar percentages of protection apply to populations recorded in Sicily, Sardinia, and Abruzzo.

The *Secale* genus is present with 33 populations all belonging to the species *Secale strictum*. A high percentage (82%) of the sites hosting these populations are in Natura 2000 Network: they mainly corresponds to SACs (89%) while the rest are ‘type C’ sites. Recorded populations are mainly located in Sicily (31) while only a few in the Marche region, 81% and 100% of these populations are under Natura 2000 protection in the two regions, respectively (**Figure 1E**).

### Distribution of *Allium, Brassica* and *Triticum* CWR species most in need of protection

Data retrieved by the National Geoportal included the distribution of 209 populations of 19 CWR species in most need of protection in Italy (‘A’ category) as defined in Ciancaleoni et al. (2021) (**Table 3**). These species belong to 3 of 5 considered genera: *Allium* (11 species), *Brassica* (7) and *Triticum* (1); indeed, none of the CWR of *Beta* and *Secale*, for which was possible to retrieve data of populations *in situ* distribution, are considered in high need of protection.

**Table 3:** List of the 19 CWR species with the highest conservation priority (‘A’ category) belonging to the genera target of this work, and their occurrence in the Italian administrative regions (Ciancaleoni et al., 2021).

Species	Endemism	Red List threat category according to Orsenigo et al. (2018; 2021) *	Number of populations	Withtin Natura 2000 (n)	Within Natura 2000 (%)
*Allium agrigentinum*	Sicily	EN	3	0	0%
*Allium franciniae*	Sicily	NT	2	1	50%
*Allium hemisphaericum*	Sicily	VU	12	10	83%
*Allium lehmannii*	Sicily	NT	15	10	67%
*Allium lopadusanum*	Sicily	EN	1	0	0%
*Allium nebrodense*	Sicily	VU	4	4	100%
*Allium obtusiflorum*	Subendemic	NT	13	6	46%
*Allium pentadactyli*	Italy	NT	2	1	50%
*Allium permixtum*	–	VU	6	5	83%
*Allium trifoliatum*	–	NT	1	0	0%
*Allium vernale*	–	VU	2	1	50%
*Brassica glabrescens*	Italy	NT	9	3	33%
*Brassica insularis*	Subendemic	–	44	33	75%
*Brassica macrocarpa*	Sicily	CR	6	6	100%
*Brassica montana*	–	–	21	18	86%
*Brassica procumbens*	–	–	3	1	33%
*Brassica rupestris*	Sicily	–	23	13	57%
*Brassica villosa*	Sicily	NT	35	9	26%
*Triticum uniaristatum*	–	–	7	4	57%

* Species included in the new Red List of the Italian endemic flora (Orsenigo et al., 2018); 2021: CR, Critically endangered; EN, Endangered; VU, Vulnerable; NT, Near Threatened. Number of populations inside and outside Natura 2000 sites is based on data retrieved from National Geoportal.

38 out of 61 (62%, *Allium*), 83 out of 141 (59%, *Brassica*) and about half of the 7 *Triticum uniaristatum* populations occur in Natura 2000 sites. When the different species of *Allium* and *Brassica* are considered, percentages are quite different ranging from 0%, in the cases of *Allium agrigentinum, A. lopadusanum* and *A. trifoliatum*, to 100% of *A. nebrodense* and from 26% of *Brassica villosa* to 100% of *B. macrocarpa* (**Table 3**).

Regarding the distribution of the priority species in the Italian administrative regions, a good agreement exists between the here presented data and those available on the ‘Portal of the Flora of Italy’ for both *Allium* and *Triticum* species (**Figures S1**, **S2**; **Supplementary Materials**, respectively). The only exceptions are *Allium hemisfericum*, known to be an endemism of Sicily although populations from different Italian regions are here reported, and *A. trifoliatum* whose distribution is here underrepresented. For *Brassica* species the same elaborations are presented in (**Figure 3** left and centre) available in the dedicated ‘case study’ section of this manuscript (see below).

### *Brassica* priority species, a case study

#### Data collection and quality control

After the application of quality control filters, the query of GBIF, Genesys and the National Biodiversity Network databases allowed to retrieve 354 records of *in situ* occurrence of the 7 *Brassica* species listed in the ‘A’ category by Ciancaleoni et al. (2021), and covered by this study, that is more than two times the number of records available in the National Geoportal for the same species (151). GBIF and the National Biodiversity Network were the most relevant databases contributing with proportions of the 0.53 and 0.39, respectively. As for the *ex situ*, 31 records from Genesys were kept after quality control.

*Brassica. montana* (100 *in situ* records), *B. insularis* (92)*, B. villosa* (74) and *B. rupestris* (61) are the species with the highest number of *in situ* records; for the same species number of *ex situ* records is quite small (7, 1, 14 and 9 respectively). Lower number of *in situ* records regarded the other 3 *Brassica* species target of this study: *B. macrocarpa* (11), *B. glabrescens* (9) and *B. procumbens* (7) (**Table 4**). Sicily (133), Sardinia (84), Liguria (58) and Tuscany (28) are the regions accounting the highest number of records; Sicily is also holding *in situ* records of the highest numbers of different species (*B. insularis, B. villosa, B. rupestris* and *B. macrocarpa*) (**Table 4**).

**Table 4:** Number *of in situ* and *ex situ* (in bracket) populations of the different CWR species belonging to the genus *Brassica* in the different Italian regions of occurrence.

Species	Calabria	Campania	Friuli-V.G.*	Lazio	Liguria	Marche	Sardinia	Sicily	Tuscany
Brassica montana**	–	–	–	4	58 (3)	15	–	–	21 (4)
*Brassica insularis*	–	2	–	–	–	–	84 (1)	6	–
*Brassica villosa*	–	–	–	–	–	–	–	74 (14)	–
*Brassica rupestris*	1	–	–	4	–	3	–	53 (9)	–
*Brassica macrocarpa*	–	–	–	–	–	–	–	11	–
*Brassica glabrescens*	–	–	9	–	–	–	–	–	–
*Brassica procumbens*	–	–	–	–	–	–	–	–	7

*Abbreviation of Friuli-Venezia Giulia; **2 populations were also recorded in San Marino.

#### Species distribution and protection status

**Figure 2:**
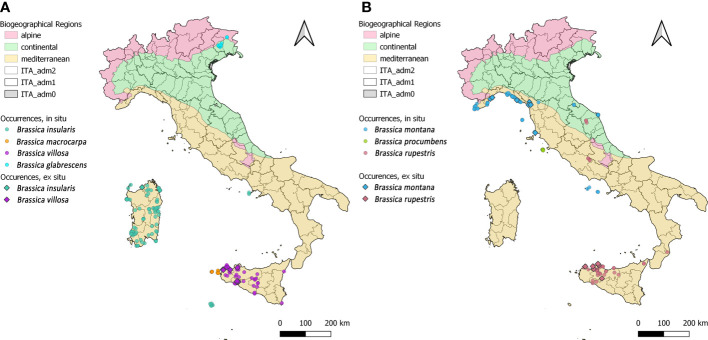
Geographical distribution of the 354 *in situ* (dots) and 31 *ex situ* (diamonds) *Brassica* species records; multiple sites with the same geographic coordinates appear as a single locality. To minimise the overlapping of occurrences, the 7 species have been clustered in two groups and represented two separated maps (**A, B**, respectively). In each map, the Italian territory is subdivided in its administrative Regions and Provinces, colours of Biogeographic Regions and species symbols are reported in the legend.

According to data in the *Brassica in situ database* developed, *Brassica montana* populations are mainly distributed along the Ligurian coast, in Lazio, in the Pontine Islands of Palmarola, Zannone, Ponza, Ventotene and S. Stefano, in Tuscany along the Gulf of Baratti and in Marche along the coast of Mount Conero; two populations are also located in the Republic of San Marino (**Figure 2**).

*Brassica insularis*, the second most common species, occurs in Sardinia, where most of the populations are located, in the Campanian Island of Ischia and in the Sicilian Island of Pantelleria. However, the records from Ischia should be confirmed not being cited among the sites of occurrence of this species in Santo et al. (2013). All populations of *Brassica villosa* are recorded in Sicily, especially along the western Tyrrhenian coast and in the province of Trapani. *Brassica rupestris* occurs in many sites in Sicily as well as in few sites in Lazio, Marche and Calabria. *Brassica macrocarpa* is only found in the Sicilian islands of Favignana, Levanzo and Marettimo. *Brassica glabrescens* and *Brassica procumbens* are only present in Friuli-Venezia Giulia and Giglio Island (Tuscany), respectively (**Figure 2**).

**Figure 3:**
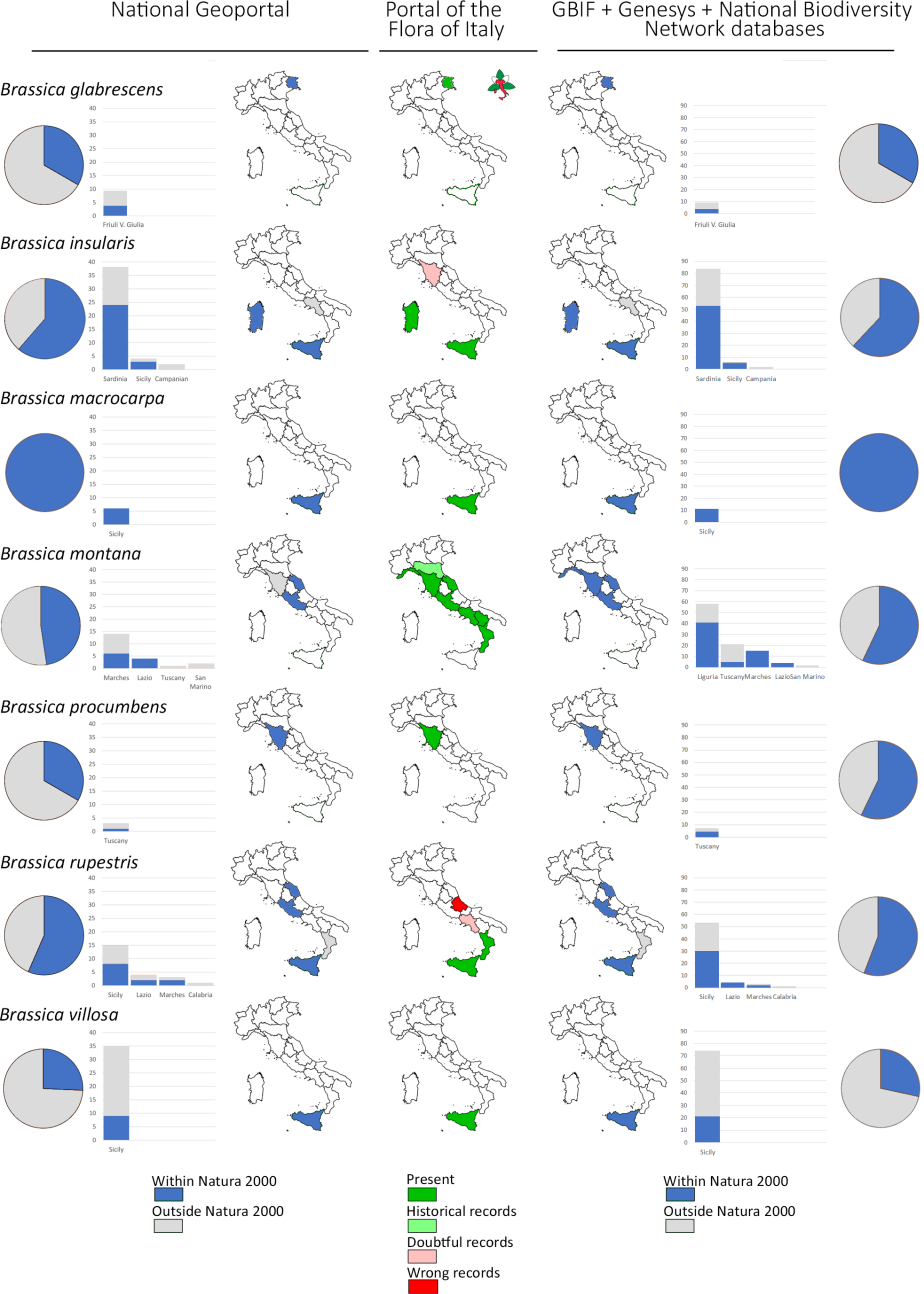
For each species in ‘A’ category of the genus *Brassica*: pie graph representation of the proportion of populations occurring within (blue) and outside (light grey) sites of the Natura 2000 network with histograms and graphical representation of the distribution in the different Italian Regions according to data in the ‘National Geoportal’ (left) and in GBIF + Genesys + National Biodiversity Network databases (right). Occurrences of each species in the different Italian Regions according to the ‘Portal of the Flora of Italy’ are also reported (center) with the corresponding colour legend.

Populations of the 7 B*rassica* CWR species are recorded in all the 3 Biogeographical regions present in Italy: mainly in the Mediterranean (325) followed by the Continental (27) and Alpine Region (2) (**Figure 2**). *Brassica insularis*, *B. macrocarpa*, *B. procumbens* and *B. villosa* only occur in the Mediterranean Region while *B. glabrescens*, *B. montana* and *B. rupestris* in more than one with the Mediterranean always prevalent. Finally, *B. glabrescens* occurs in Alpine and Continental Regions (**Figure 2**). According to data collated from GBIF, Genesys, and the National Biodiversity Network database the percentage of sites included in the Natura 2000 Network, as well as their distribution in the different Italian regions, resembles quite well those reported in both ‘National Geoportal’ and ‘Portal of the Flora of Italy’ (**Figure 3**). The only exceptions were *B. montana*, and *B. rupestris*, for the distribution in the administrative regions, and *B. procumbens* for the percentage of sites occurring in Natura 2000 Network (**Figure 3**).

#### Land cover and land use

**Figure 4:**
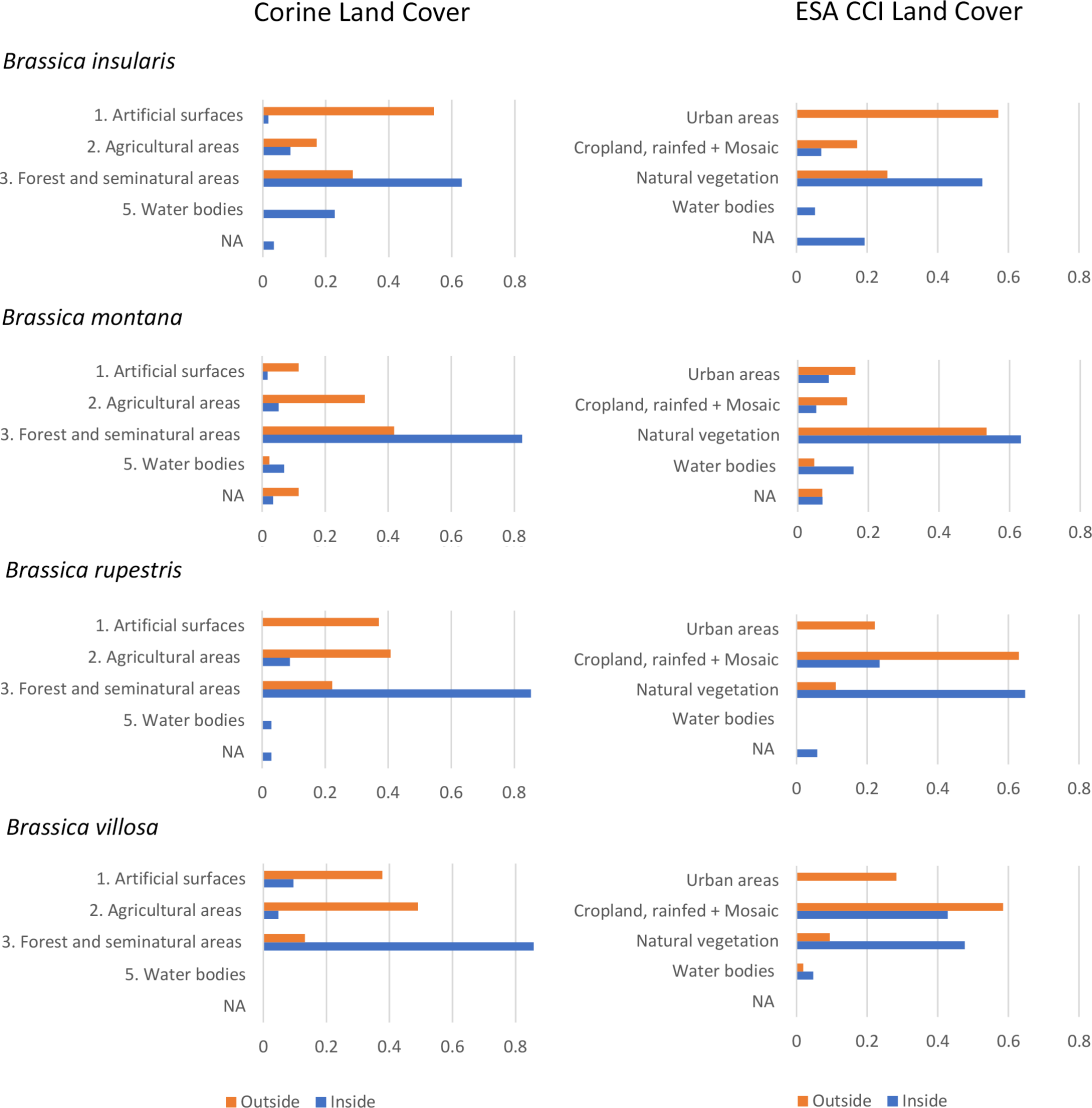
LCLU of site holding priority *Brassica* CWR populations located outside (orange) and inside (blue) Natura 2000 network according to Corine (left) and ESCA CCI (right) classifications.

**Figure 5:**
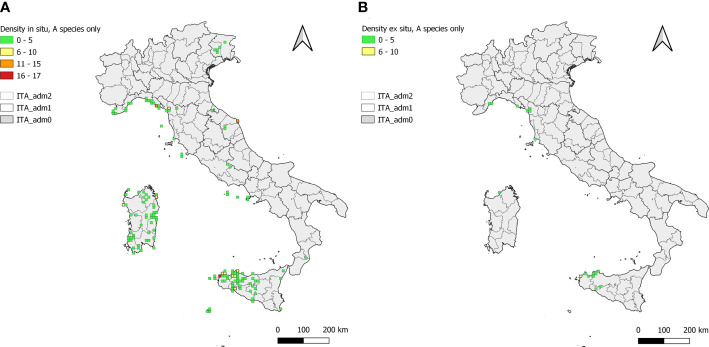
Density of *in situ* **(A)** and *ex situ* **(B)** *Brassica* CWR population occurrences in cells of side 10 × 10 km. In each map, the Italian territory is subdivided in its administrative Regions and Provinces; cell colours are according to the legend.

LCLU analysis results are summarised in **Figure 4**. Most of the sites holding *Brassica insularis* populations outside Natura 2000 areas are quite anthropized and classified as *artificial surfaces* + *agricultural areas* (proportion of 0.74, Corine) or *urban areas* + different cropland categories (0.71, ESA CCI). Vice versa, LCLU of sites holding CWR populations of the same species located in Natura 2000 sites mainly corresponds to *forest and seminatural areas* (0.63, Corine) or, similarly, to several categories of sites characterised by the occurrence of natural vegetation (0.53, ESA CCI). Outside Natura 2000 areas, only a low proportion of the sites (0.26) corresponds to *forest and seminatural areas* or to sites characterised by natural vegetation according to both classifications (**Figure 4**, *B. insularis*). Sites hosting populations of *Brassica rupestris* and *B. villosa* show similar characteristics. Outside Natura 2000 areas, anthropized sites account for high proportions: 0.78 and 0.87 (Corine) and 0.85 and 0.87 (ESA CCI), for each of the two species, respectively; while those characterised by natural or seminatural vegetation account for a considerably lower proportion: 0.22 and 0.13 (Corine) and 0.11 and 0.09 (ESA CCI), respectively. Except for ESA CCI classification of *B. villosa* sites − fairly shared between anthropized and natural/seminatural LCLU categories − sites located inside Natura 2000 areas are mainly natural/seminatural for both species and classification systems (**Figure 5**, *B. rupestris* and *B. villosa*).

As for *B. montana*, LCLU of sites in Natura 2000 Network is mainly natural: *forest and seminatural areas* (0.82, Corine) prevail, or different categories of sites characterised by the occurrence of natural vegetation (0.63, ESA CCI). Outside Natura 2000 the percentage of strongly anthropized sites is moderate, proportions of 0.44 (Corine) and 0.30 (ESA CCI) while a moderate proportion shows a natural or seminatural LCLU (0.42 and 0.53 according to Corine and ESA CCI, respectively) (**Figure 4**, *B. montana*).

#### *In situ* vs *ex situ* density analyses

A total of 143 and 17 cells (10 × 10 km) include ≥1 *Brassica in situ* and *ex situ* records, respectively (**Figure 5**). Cells accounting for 1 occurrence were prevalent both *in situ* (proportion of 0.48) and *ex situ* (0.71) followed by those accounting for 2 and 3 occurrences (0.20 and 0.12 and 0.12 and 0.06 in *in situ* and *ex situ*, respectively). The highest *in situ* record densities were recorded in Sicily, mainly between Trapani and Erice (Trapani province), Liguria, between Monteresso al Mare and Corniglia (Spezia) and Marche, at Conero (Ancona) (**Figure 5A**). Densities were generally lower for *ex situ* records with the only cell characterised by values >5 records located in Sicily (Trapani province) (**Figure 5B**).

Gaps between *in situ* and *ex situ* populations occurrence are quite spread across the Italian territory with few exceptions represented by isolated and restricted areas of the Ligurian coast and of Sicily and Sardinia (**Figures 5A, B**). In terms of *in situ* and *ex situ* population density difference most relevant gaps regarded *Brassica insularis* in Sardinia, where populations are scattered in many sites across the island while are almost completely missing in *ex situ* conservation facilities, *B. montana* at Conero (Marche) and Spezia (Liguria) and *B. villosa* around Trapani in Sicily (**Figures 5A, B**).

## Discussion

We collated and analysed population distribution data of CWR species belonging to the world priority genera *Allium, Beta, Brassica, Secale* and *Triticum* in Italy to help assessing their present conservation status and provide a data-driven basis for planning future conservation actions. Even if occurrence is only one of the information needed to assess the conservation status of a species, such information is often given at quite low spatial resolution (*e.g.* administrative Region’s level) and mainly as species presence *vs* absence. In addition, when available, *in situ* populations occurrence data are frequently outdated, not verified, and negatively affected by limited spatial resolution. Contrarily, by taking advantage of data available in the Italian National Geoportal, we profitably generated and analysed a high-quality dataset that allowed to provide a first comprehensive overview of target species distribution across the Italian territory with respect to their occurrence in the Natura 2000 network; such information is of great utility to identify critical points and direct the best management decisions. Indeed, evaluating the extent of populations within the protection network was our main goal considering the role that Natura 2000 can have in increasing CWR *in situ* conservation. In addition, for species in most need of protection belonging to the genus *Brassica*, we generated a solid dataset of information by applying a strict quality control to data available in different online databases (*i.e.* GBIF, Genesys and National Biodiversity Network); this approach allowed for a more detailed analysis of the distribution of target species, involving more than two times the number of occurrences, and for some comparisons between the different data sources. To provide useful information for an active conservation at both National and Regional level, population distribution was also assessed considering the territory subdivision in the different Italian Administrative Regions.

Based on the ‘National Geoportal’ only, information on 1,996 populations of the six considered genera were retrieved, about 67% of which are included in Natura 2000 sites. Except for the genus *Allium*, homogeneously present in all Italian regions, the target species appear to be mainly concentrated in Central and Southern Italy especially when species most in need of protection are considered. Considering the CWR in each genus, percentages of sites hosting CWR diversity located in Natura 2000 were quite high for *Secale* (82%) as well as for the two most numerous genera *Allium* and *Brassica* (68% each, respectively); slightly lower values were obtained for *Beta* (53%) and *Triticum* (40%). In a recent study Rubio Teso et al. (2020) analysed the presence of CWR populations in the Natura 2000 in the European Union and United Kingdom. Considering only priority species with high quality distribution data, the authors showed that the 22% and about the 40% of *Allium* and *Brassica* populations are in Natura 2000 sites, respectively. The main reasons for the lower overall percentages obtained by Rubio Teso and colleagues, in comparison with the here reported percentages, could be due to the considered species, selected using different prioritization criteria, as well as to the wider geographical area considered in the work of Rubio Teso et al. (2020). As for *Triticum*, even if number of records and target species were higher in the same work by Rubio Teso and colleagues, the calculated percentage of populations experiencing protection was quite similar: 40% *vs.* 37% in this and the cited work, respectively. As for the species belonging to *Beta* and *Secale*, data comparison between the two studies was not possible due to the great area of distribution of *B. vulgaris* subsp. *maritima*, recorded from the North Atlantic coasts to Morocco and in all countries in the Mediterranean basin, that would have made the comparison results quite biased, and to the limited number of records available for *Secale strictum*. At this regard it should be noted that here reported distribution data of *Beta vulgaris* subsp. *maritima*, as well as of *Brassica fruticulosa*, appear to be an underrepresentation of the actual occurrence of the two species.

When only priority species in Italy are considered, *i.e.* those belonging to the ‘A’ category in Ciancaleoni et al. (2021), *Allium* (11 species), *Brassica* (7) and *Triticum* (1) are the genera of highest interest among the 5 here considered.

The number of sites hosting populations of *Allium* ‘A’ category CWR species − all at risk according to Orsenigo and colleagues (2021) − are quite different and limited in many cases: less than 10 for *Allium agrigentinum, A. franciniae, A. lopadusanum, A. nebrodense, A. pentadactyli, A. permixtum, A. trifoliatum* and *A. vernale* and few more for the other species. Considering the reported numbers, urgent actions favouring the survival of these populations *in situ* are needed. At this regard it should also be considered that with the only exception of *A. trifoliatum*, all these species are endemisms of Italy, or even of the Sicily region, that makes their protection even most urgent.

As for populations of ‘A’ category CWR species of *Brassica*, the percentage of populations occurring in Natura 2000 sites, as well as their distribution in the Italian regions, was quite similar in the different used databases. With the only exceptions of *Brassica insularis* and *B. montana*, such percentages are also similar to those reported by Rubio Teso and colleagues (2020) for the other species common to the two studies: *B. macrocarpa* (100%), *B. rupestris* (57%) and *B. villosa* (41%). This was not unexpected considering that: i) data collection and quality control procedures were quite similar and ii) some of the species are almost exclusively present in the Mediterranean part of Italy and in its major islands (Sicily and Sardinia). In fact, according to data available in EURO+MED database, *B. insularis* is an endemic Mediterranean species that, in Europe, only occurs in Corsica, Sardinia and Sicily with Malta; *B. macrocarpa* occurs in Sicily and Malta; *B. rupestris* in Croatia, Italy and Sicily with Malta and *B. villosa* only in Sicily with Malta. *B. montana* only is characterised by a wider distribution occurring in France and Monaco (France), Italy, Poland, Spain with Gibraltar and Andorra (Spain) (Tutin et al., 1964). Considering the high value of the related crops (*e.g.* cabbage, cauliflower, broccoli) for the country economy, traits of interest for plant breeding (Ciancaleoni et al., 2021; Perrino and Wagensommer, 2021) and wild species distribution, conservation of *Brassica* CWR is particularly important for Italy that is the main reason behind their selection for the reported case study. Noteworthy, a direct use has been reported for different species of *Allium* and *Brassica* genera (*e.g. Brassica fruticulosa*, *B. incana*, *B. montana* and *B. procumbes*) (Ciancaleoni et al., 2021). About the need of greater protection of *Brassica* CWR in Italy, it should be noted that according to our results populations outside Natura 2000 sites mainly occur in anthropized areas which makes difficult their future survival opportunities if no conservation actions are taken. Finally, it should also be considered that, from both conservation and use perspective, not the species but single populations within a species are relevant, since single populations may retain specific genetic arrangements and traits of value for crop breeding. At this regard, it has been already shown that quite a lot of differentiation exists even among Italian *Brassica incana* populations located in the same geographical area (Ciancaleoni et al., 2018). With the single exception of *B. macrocarpa*, populations not included in sites of the Natura 2000 Network exist for all the considered *Brassica* species most in need of protection.

Due to its close relationship with cultivated wheats, *Triticum* genus has been intensively studied; geographical distribution, ecology and vulnerability of its species in Italy have been recently updated (Perrino et al., 2014 and references therein). Wheat CWRs conservation is quite relevant since it has been suggested that they still retain traits potentially useful to increase technological and nutritional quality (*e.g.* grains micro‐nutrient quantity, including Fe and Zn) and for resistance/tolerance to both biotic and abiotic stresses (Perrino et al., 2014) that are particularly needed under the actual climate change scenario; for an updated and comprehensive review on the topic see Sharma et al. (2021). Listed in Annex I of the FAO International Treaty on Plant Genetic Resources for Food and Agriculture (ITPGRFA), many of the species of this genus present in Italy are at low risk (LC) according to the Red Lists (Bilz et al., 2011). However, *Triticum uniaristatum* is considered vulnerable (VU) in the updated Red List of vascular flora native to Italy (Orsenigo et al., 2021). The here reported distribution of populations of *T. uniaristatum*, as well as its presence in the different Italian regions, is coherent with what is known for the species. The limited distribution, together with other risk factors, and grazing in particular, has been suggested among the factors making this species particularly vulnerable (Perrino et al., 2014). With only seven population records, the four located within Natura 2000 could be crucial for the conservation of this very rare *taxon* in Italy.

Calculated on a large dataset of punctual geographical distribution of 1,996 populations belonging to 60 different CWR species, the here presented data show that the Natura 2000 Network has a potentially relevant role in favouring CWR *in situ* conservation in Italy and Europe. Additionally, although populations located in Natura 2000 sites already experience a passive safeguard, an active protection including, at least, a regular demographic, ecological and geographic monitoring of the existing populations should be pursued by directing specific funds and dedicated action plans. A future, desirable active *in situ* conservation of European priority CWR in Natura 2000 sites could provide an important added value to the network through a contribution to biodiversity conservation as well as to food security (Rubio Teso et al., 2020).

A step forward in protecting CWR in Italy could also involve the institution of new ‘genetic reserves’, *i.e.* of sites for the management and monitoring of genetic diversity of natural wild populations within defined areas designated for active, long-term conservation (Iriondo et al., 2012; Maxted et al., 2015). According to evidence from this study, areas around Trapani and Palermo (Sicily) emerge as interesting candidates hosting multiple populations of different *Brassica* CWR species most in need of protection (*i.e. Brassica rupestris* and *B. villosa*). The Natura 2000 sites already present in the area like *Monte Cofano, Capo San Vito e Monte Sparagio* (type A, ITA010029), *Raffo Rosso, Monte Cuccio e Vallone Sagana* (type B, ITA020023) or *Montagna Longa, Pizzo Montanello* (the type B, ITA020021) could be an excellent starting point for the institution of the genetic reserves so that to guarantee an active safeguard of such important plant genetic resources. To maximise benefits retrievable by the institution of new genetic reserves, the co-occurrence of populations of different CWR species should be considered using approaches similar to those described in the present study for *Brassica*.

We also demonstrated that many populations of CWR related to crop species of great value occur outside protected areas. Since these populations with their diversity could usefully contribute to sustainable *in situ* conservation, it is urgently needed to take action to establish a certain level of protection even in these areas. Although the institution of further protected areas might not be feasible or affordable, good results might be achieved by means of the ‘Out of Reserve Management’ approach (Hale and Lamb, 1997) through the implementation and application of management plans to reduce the level of risk and the related threats for these valuable CWR populations. The urgent need of appropriate ‘Out of Reserve Management’ plans is also suggested by the results of the LULC analysis that showed a strong anthropization of sites hosting CWR species not located in the Natura 2000 Network. In this regard it has recently become clear that anthropogenic habitats, especially agro-ecosystems, must be incorporated into conservation strategies to achieve a successful conservation of biodiversity and associated ecosystem services (Millennium Ecosystem Assessment, 2005; Scherr and McNeely, 2008). Several researches on the attributes that make croplands and agro-landscapes useful for biological conservation have been also produced (Mellink et al., 2017 and references therein). Finally, e*x situ* conservation could also play a significant role in securing the survival of target populations, and the access to their diversity for research as well as breeding activities. Historically collection and *ex situ* conservation of CWR species have been overlooked with the consequence that these species are underrepresented in most of the genebank collections (FAO, 2010 and references therein). If presented data in the one hand confirm the alarming scenario for the *Brassica* species in most need of protection in Italy, whose *in situ* distribution is not adequately represented *ex situ*, in the other hold valuable information to plan new collection missions for these species.

## Conclusions

In this study we presented the results of the first comprehsnsive analysis of the distribution in Italy of CWR species belonging to genera that are of key relevance at European, as well as global level. Considering that Italy is placed at the core of the Mediterranean biodiversity hotspot, and that important CWR species are endemism of the Country, our results are quite alarming. Indeed, data shows that several wild progenitors of crops of great relevance are in rather precarious conditions *in situ*; this is mainly due to the limited knowledge and/or number of populations of the most threatened species. Discriminating between these two cases, and, when possible, increase the efforts for *taxa* detection across the territory, is crucial to develop protection actions that are effective and commensurate with the real needs of biodiversity conservation. On the other hand we also got evidence that holding populations of different CWR species, the Natura 2000 Network have a significant role in favouring CWR *in situ* conservation in Italy, as well as in the entire EU, that can be further increased if an ‘active’ protection will be pursued and finally achieved. Finally, according to presented data, further efforts should be also dedicated to protect CWR populations outside protected areas through a wise application of the ‘Out of Reserve Management’ approach and *ex situ* conservation actions.

## Data availability statement

Publicly available datasets were analyzed in this study. This data can be found here: http://geoviewer.nnb.isprambiente.it/mapreacter.

## Author contributions

LR: conceptualization, methodology, formal analysis, investigation, data curation, writing – original draft, writing– review & editing, visualization. CZ: formal analysis, writing. DG: writing– review & editing, funding acquisition. VN: research planning and conceptualization, methodology, investigation, writing – original draft, writing– review & editing, supervision, funding acquisition. All authors contributed to the article and approved the submitted version.
