# Supplementary material for: In situ occurrence and protection of crop wild relatives in Italian sites of natura 2000 network: Insights from a data-driven approach
Source: Front Plant Sci. 2022 Dec 22;13:1080615. doi: 10.3389/fpls.2022.1080615 (PMC9814127; doi:10.3389/fpls.2022.1080615)
Supplement: Supplementary file 1 [file DataSheet_1.docx]

In situ occurrence and protection of crop wild relatives in Italy: insights from a data-driven approach

Lorenzo Raggi, Cecilia Zucchini, Daniela Gigante and Valeria Negri

Dipartimento di Scienze Agrarie, Alimentari e Ambientali (DSA3), Università degli Studi di Perugia, Perugia, Italy


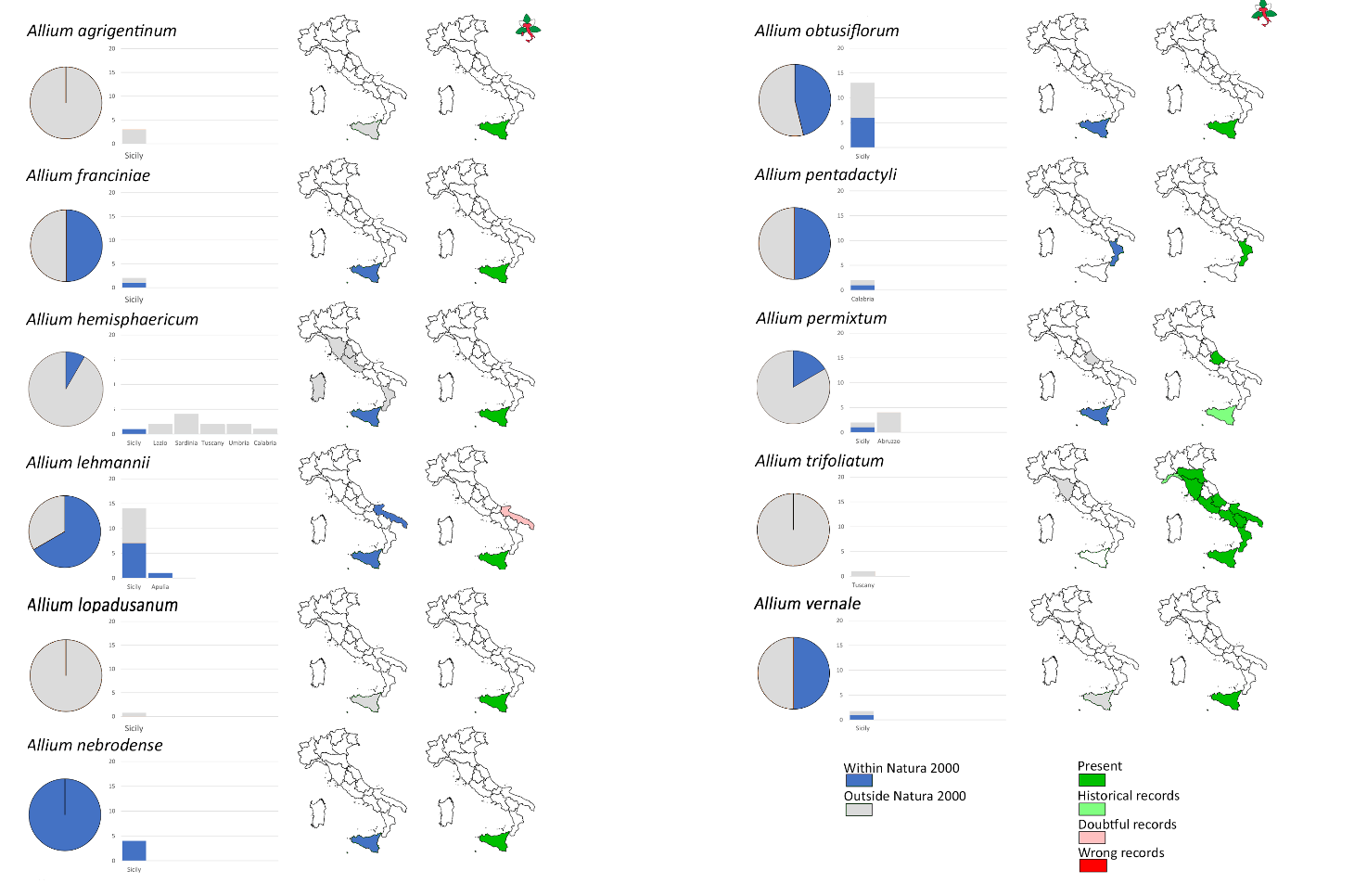


Figure S1. From left to right, for each species of the genus *Allium* in “A” category according to Ciancaleoni et al. (2021): pie graph representation of the proportion of populations occurring within (light blue) and outside (light grey) sites of the Natura 2000 network with histograms and graphical representation of the distribution in the different Italian Regions. Colours indicating the presence (or not) of each species in the different Italian Regions are according to the ‘Portal of the Flora of Italy’ (also reported in the legend).


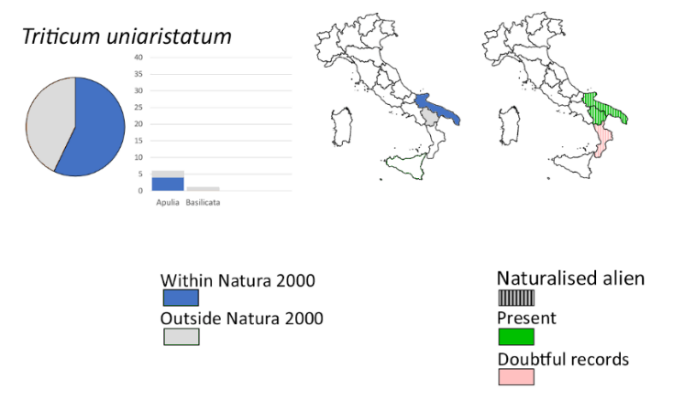


Figure S2. From left to right, pie graph representation of the proportion of populations of *Triticum uniaristatum* (“A” category according to Ciancaleoni et al. (2021)) occurring within (light blue) and outside (light grey) sites of the Natura 2000 network with histograms and graphical representation of the distribution in the different Italian Regions. Colours indicating the presence (or not) of each species in the different Italian Regions are according to the ‘Portal of the Flora of Italy’ (also reported in the legend).
